# Negative COVID-19 Vaccine Information on Twitter: Content Analysis

**DOI:** 10.2196/38485

**Published:** 2022-08-29

**Authors:** Niko Yiannakoulias, J Connor Darlington, Catherine E Slavik, Grant Benjamin

**Affiliations:** 1 School of Earth, Environment and Society McMaster University Hamilton, ON Canada; 2 School of Geography and Environmental Management University of Waterloo Waterloo, ON Canada; 3 Center for Science Communication Research School of Journalism and Communication University of Oregon Eugene, OR United States; 4 Department of Economics University of Toronto Toronto, ON Canada

**Keywords:** vaccine acceptance, vaccine hesitancy, Twitter, health communication, COVID-19, social media, infodemiology, misinformation, content analysis, sentiment analysis, vaccine misinformation, web-based health information

## Abstract

**Background:**

Social media platforms, such as Facebook, Instagram, Twitter, and YouTube, have a role in spreading anti-vaccine opinion and misinformation. Vaccines have been an important component of managing the COVID-19 pandemic, so content that discourages vaccination is generally seen as a concern to public health. However, not all negative information about vaccines is explicitly anti-vaccine, and some of it may be an important part of open communication between public health experts and the community.

**Objective:**

This research aimed to determine the frequency of negative COVID-19 vaccine information on Twitter in the first 4 months of 2021.

**Methods:**

We manually coded 7306 tweets sampled from a large sampling frame of tweets related to COVID-19 and vaccination collected in early 2021. We also coded the geographic location and mentions of specific vaccine producers. We compared the prevalence of anti-vaccine and negative vaccine information over time by author type, geography (United States, United Kingdom, and Canada), and vaccine developer.

**Results:**

We found that 1.8% (131/7306) of tweets were anti-vaccine, but 21% (1533/7306) contained negative vaccine information. The media and government were common sources of negative vaccine information but not anti-vaccine content. Twitter users from the United States generated the plurality of negative vaccine information; however, Twitter users in the United Kingdom were more likely to generate negative vaccine information. Negative vaccine information related to the Oxford/AstraZeneca vaccine was the most common, particularly in March and April 2021.

**Conclusions:**

Overall, the volume of explicit anti-vaccine content on Twitter was small, but negative vaccine information was relatively common and authored by a breadth of Twitter users (including government, medical, and media sources). Negative vaccine information should be distinguished from anti-vaccine content, and its presence on social media could be promoted as evidence of an effective communication system that is honest about the potential negative effects of vaccines while promoting the overall health benefits. However, this content could still contribute to vaccine hesitancy if it is not properly contextualized.

## Introduction

Major social media platforms, such Facebook, Instagram, Twitter, and YouTube, have been studied for their role in spreading anti-vaccine opinion and misinformation in recent years [1-3]. Evidence suggests that this content may be responsible for lower vaccine coverage in some populations [4-7]. Several processes could explain how content on these platforms influences opinions about vaccines. The simplest explanation is that information directly changes beliefs and behavior; for example, misinformation may lower the acceptance of vaccines by influencing what people believe to be true [8]. Another explanation is that social media is effective at mobilizing the engagement of like-minded individuals that reinforces anti-vaccine perspectives [9]. Another mechanism is psychological reactance—information that appears to threaten freedom of choice (such as vaccine certification) can motivate resistance to recommended behaviors (such as vaccination) [10].

Vaccine hesitancy—a concern toward vaccines that can lead to a delay or rejection of recommended scheduled immunization—is the product of personal experience, knowledge, and structural factors that influence individual trust in the efficacy and safety of vaccines [11,12] and, therefore, could emerge without exposure to misinformation or explicitly anti-vaccine sentiment. For example, the media reporting of genuine adverse reactions to vaccines or infections among vaccinated individuals could increase concerns in the value of vaccination without being factually untrue or expressing an anti-vaccine opinion. In this study, we use the concept of negative vaccine information (NVI) to describe content that characterizes vaccines or vaccination in a negative way without also expressing anti-vaccine sentiment. NVI includes descriptions of possible side effects, vaccine quality concerns, vaccine contamination, statistics on morbidity or mortality associated with vaccination, and negative personal experiences of vaccination.

NVI may be an important part of the communication of vaccine information, since an individual’s choice to vaccinate often involves a comparison of health risks to health benefits. Adverse events following vaccination—while uncommon—can occur, and vaccination can be associated with other consequences of personal concern [13]. Provided that vaccination remains a choice, the communication of information about adverse events and vaccine efficacy is necessary to empower people to make personally satisfying decisions. This information may also increase the credibility of the information provider [14], and although it could lead to greater vaccine hesitancy for some people in the short term, it could also increase the trust of public authorities in the long term [15].

Conversely, NVI may be harmful when it lacks contextual background and contributes to greater concern and hesitancy [16]. In the early days of COVID-19 vaccines, the lack of knowledge would have made normal subjective interpretations about vaccine safety and value more challenging [17]. NVI could have been the first information that some people encountered on the web, providing simple accounts of discomfort or adverse reactions that are easier to process than the statistics showing that the balance of evidence is in favor of vaccination. This information, when combined with a number of well-known cognitive biases, may have contributed to some of the vaccine hesitancy during the early period of COVID-19 vaccination [18].

The primary objective of this research was to understand the prevalence and characteristics of NVI on Twitter during the first 4 months of 2021. This time period was chosen because it follows a shift in policy from Twitter and other major social media platforms to address growing concerns about COVID-19 vaccine misinformation [19-21]. There is a consensus that this shift resulted in a decline in anti-vaccine misinformation on these platforms as well as an open question as to whether these changes in policy removed most NVI content. This time period was also marked by the widespread discussion of vaccine brand preference, vaccine nationalism, and early concerns of differences in adverse reaction risk [22], all of which could be a part of various NVI narratives. In this research, we estimated the prevalence of anti-vaccine content and NVI by time, geography, and vaccine developer to understand the degree to which changes in policy by social media outlets may have impacted the availability of both anti-vaccine content and NVI to the public.

## Methods

### Data Collection

We used the Twitter API to collect data on vaccines and vaccination between December 23, 2020, and April 30, 2021, using the *rtweet* package [23]. An R script was used to automate a search of tweet text, up to 18,000 tweets every 20 minutes, matching the search condition in stage 1 (Table 1). After excluding retweets, this stage yielded a total of 7,827,949 tweets. Next, the text of each tweet was searched to identify and retain only tweets referring to 1 or more of the search terms in stage 2, resulting in 785,107 tweets. In stage 3, each tweet’s geocoded country field, location field, and description field were searched, keeping only those tweets with at least one of the stage 3 search terms in at least one of these fields. Tweets were then georeferenced to Canada, the United States, or the United Kingdom based on the geographic information associated with the tweet author’s country, location, or description field. These countries were chosen because they initiated vaccination at similar times (in late 2021) and had a sufficient volume of English-language tweets related to vaccine to facilitate analysis. For some tweets, the country, location, and description fields indicated more than 1 country—the United Kingdom, Canada, and the United States—as a location. In these instances, the order of dominance was country, location, and description. For example, if a tweet author had “Canada” in location and “United States” in their description, they were assigned to “Canada.” Occasionally, the location and description fields contained more than 1 country. In these cases, the tweets were deleted. In addition, only tweets whose authors had at least 1 follower and only English-language tweets were retained. This process resulted in 217,954 tweets, which was the final sampling frame used in this study.

**Table 1:** Search criteria.

Stage	Search criteria
Stage 1	(“covid” OR “coronavirus” OR “corona” OR “sars-cov-2” OR “sarscov2”) AND (“vaccine” OR “vaccination” OR “vaccinated” OR “shot” OR “inoculate” OR “inoculation” OR “inoculated” OR “immunize” OR “immunized” OR “immunization”)
State 2	(“astrazeneca” OR “astrazeneca” OR “azd1222” OR “covishield” OR “vaxzervia” OR “oxford-astra-zeneca” OR “oxford-astrazeneca” OR “oxfordastrazeneca” OR “pfizer” OR “tozinameran” OR “BNT162b2” OR “biontech” OR “pfizer-biontech” OR “fosun-biontech” OR “pfizerbiontech” OR “fosunbiontech” OR “moderna” OR “mrna-1273” OR “cx-024414” OR “tak919” OR “cx024414” OR “tak919” OR “mrna1273” OR “sputnik” OR “sputnikv” OR “gam-covid-vac” OR “gamcovidvac” OR “sinopharm” OR “bbibpcorv” OR “bbibpcorv” OR “johnsonandjohnson” OR “johnson&johnson” OR “janssen” OR “ad26.cov2,s” OR “jnj-78436735” OR “ad26covs1” OR “vac31518” OR “sinovac” OR “coronavac” OR “picovacc” OR “covaxin” OR “bbv152” OR “whole-virioninactivated” OR “wholevirioninactivated” OR “bharat” OR “novavax” OR “nvx-cov2373” OR “nvxcov2373” OR “sars-cov-rs” OR “covovax”)
Stage 3	(“Canada” OR “Canadian” OR “British Columbia” OR “Alberta” OR “Saskatchewan” OR “Manitoba” OR “Ontario” OR “Quebec” OR “New Brunswick” OR “Nova Scotia” OR “Prince Edward Island” OR “Newfoundland” OR “Yukon” OR “Northwest Territories” OR “Nunavut” OR “U.S.A.” OR “USA” OR “United States of America” OR “United States” OR “American” OR “Alabama” OR “Alaska” OR “Arizona” OR “Arkansas” OR “California” OR “Colorado” OR “Connecticut” OR “Delaware” OR “Florida” OR “Georgia” OR “Hawaii” OR “Idaho” OR “Illinois” OR “Indiana” OR “Iowa” OR “Kansas” OR “Kentucky” OR “Louisiana” OR “Maine” OR “Maryland” OR “Massachusetts” OR “Michigan” OR “Minnesota” OR “Mississippi” OR “Missouri” OR “Montana” OR “Nebraska” OR “Nevada” OR “New Hampshire” OR “New Jersey” OR “New Mexico” OR “New York” OR “North Carolina” OR “North Dakota” OR “Ohio” OR “Oklahoma” OR “Oregon” OR “Pennsylvania” OR “Rhode Island” OR “South Carolina” OR “South Dakota” OR “Tennessee” OR “Texas” OR “Utah” OR “Vermont” OR “Virginia” OR “Washington” OR “West Virginia” OR “Wisconsin” OR “Wyoming” OR “UK” OR “U.K.” OR “England” OR “Wales” OR “Northern Ireland” OR “Scotland” OR “United Kingdom” OR “British” OR “Scottish” OR “Welsh” OR “English”)

### Data Classification

In total, 9000 tweets were randomly sampled (with replacement) from this sampling frame. After training on a separate sample of 200 tweets, all 4 authors read through mutually exclusive subsamples of 8800 tweets and coded every tweet in which the text contained negative information or sentiment about vaccines as “1”; otherwise, the tweets are coded as “0.” This criterion includes statistics or reports of adverse reactions, personal statements about adverse reactions, skepticism toward vaccine developers, policy decisions to stop or delay the administration of vaccines, expressions of concern about the vaccines, and implicitly or explicitly anti-vaccine tweets. After these tweets were coded, the authors went through the tweets coded as “1” and distinguished between those that were implicitly or explicitly anti-vaccine and those that were not. Tweets that were not anti-vaccine were classified as NVI, and the remainder were classified as anti-vaccine. Textbox 1 illustrates examples of anti-vaccine and NVI tweets. Each coder classified a random sample of 300 of the same tweets to compare interrater reliability using Krippendorff α [24]. Finally, after all the above coding was completed, each tweet author was coded into 1 of 5 types: media, medical and health, government, other, or restricted/closed account. Due to an error in data extraction early in the study, tweets were excluded if they occurred outside the period from January 6 to April 30, 2021. The final data set included 7306 tweets exclusive of the 300 tweets used to compare agreement between data coders, which were not used in the analysis.

**Examples of anti-vaccine and negative vaccine information (NVI) tweets.**

**Anti-vaccine tweets**

- “Israelis got facial paralysis after having received the Pfizer Covid vaccine. This vaccine is anything but safe. It’s not Covid which is threatening the public health. It’s the Pfizer vaccine”
- “Please listen and share widely esp. with authorities. Moderna/Pfizer in highly deceptive, harmful medical practice re covid “vaccine” (in fact ‘gene therapy technology)...”
- “These vaccines are not safe for everyone! Do not be peer pressured into destroying your life over this!”
- “An experimental vaccine using experimental technology. And in the case of Moderna, a company with no prior pharmaceutical, much less vaccine track record. Shame on you if you don’t protect the people against mandatory Covid vaccine by employers and businesses.”
- “6 people died after taking the Pfizer vaccine, I didn’t look into Moderna yet. Since There are known treatments for covid, why would anyone want to take a vaccine With unknown long term side effects especially given how extremely high covid’s survival rate is?”

**NVI tweets**

- “I’m curious of your age bracket. I’m mid sixties and received my 2nd Pfizer on the 3rd. Just the arm pain. My SIL late 30’s contracted covid-19 got the first moderna shot and was down for 2 days. Point being I believe the youth have more side effects bc of better immune sys.”
- “The European Medicines Agency (EMA) said on Wednesday it had found a possible link between AstraZeneca’s coronavirus vaccine and reports of very rare cases of blood clots in people who had received the shot.”
- “Well i guess i join the many that experience side effects of the 2nd vaccine. 12 hours later. Aches, chills and a horrendously sore arm. Started to feel better after a few hours. Now im just sore.”
- “AstraZeneca Concerns Throw Europe’s Covid-19 Vaccine Rollout Into Deeper Disarray”
- “Moderna says possible allergic reactions to COVID-19 vaccine under investigation”

## Results

Of the 300 tweets coded by all authors for the purpose of measuring between coder reliability, 79% (n=237) had full agreement across all content coders on NVI (Krippendorff α=0.63) and 80% (n=240) had full agreement across all content coders on anti-vaccine content (Krippendorff α=0.25).

Of the 7306 tweets, 131 (1.8%) were coded as anti-vaccine and 1533 (21%) were coded as NVI. Table 2 shows the anti-vaccine and NVI tweet frequencies by author type. Due to the small number of anti-vaccine tweets and the relatively low level of interrater agreement of anti-vaccine tweets, no further analysis of these data is shown, and all remaining analysis excluded anti-vaccine tweets.

**Figure 1:**
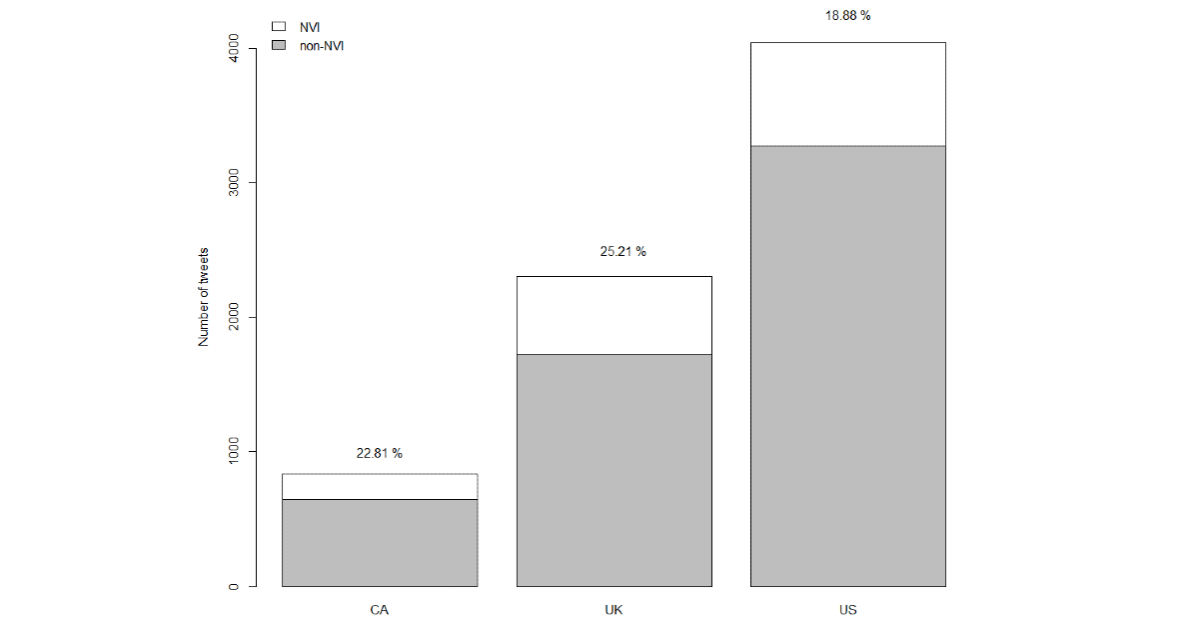
Frequency of NVI and non-NVI tweets by country. Percentages are the fraction of tweets in a country that have NVI. CA: Canada; NVI: negative vaccine information; UK: United Kingdom; US: United States.

For the 1533 NVI tweets, 37.9% (n=581) originated from the United Kingdom, 49.7% (n=762) originated from the United States, and 12.4% (n=190) originated from Canada. The total number of tweets and percentage of NVI tweets by geography are shown on Figure 1. Pairwise *z* tests of differences in the percentage of NVI tweets in this figure suggest that the apparent difference between Canada and the United Kingdom could be due to chance (*P*=.23), although the differences were statistically significant in the comparison between Canada and the United States (*P*=.01) and between the United Kingdom and the United States (*P*<.001).

**Figure 2:**
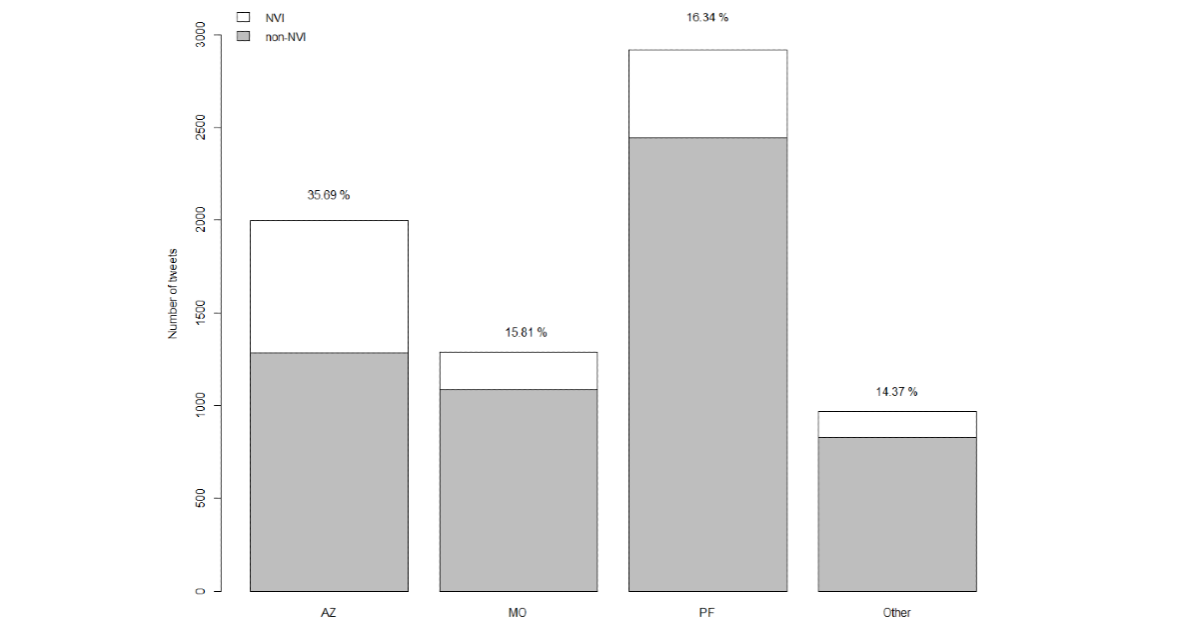
Frequency of NVI and non-NVI tweets by COVID-19 vaccine developers. Numbers inside bars are percentages of tweets that are NVI. AZ: Oxford/AstraZeneca; MO: Moderna; NVI: negative vaccine information; Other: any other COVID-19 vaccine developer; PF: Pfizer-BioNTech.

**Figure 3:**
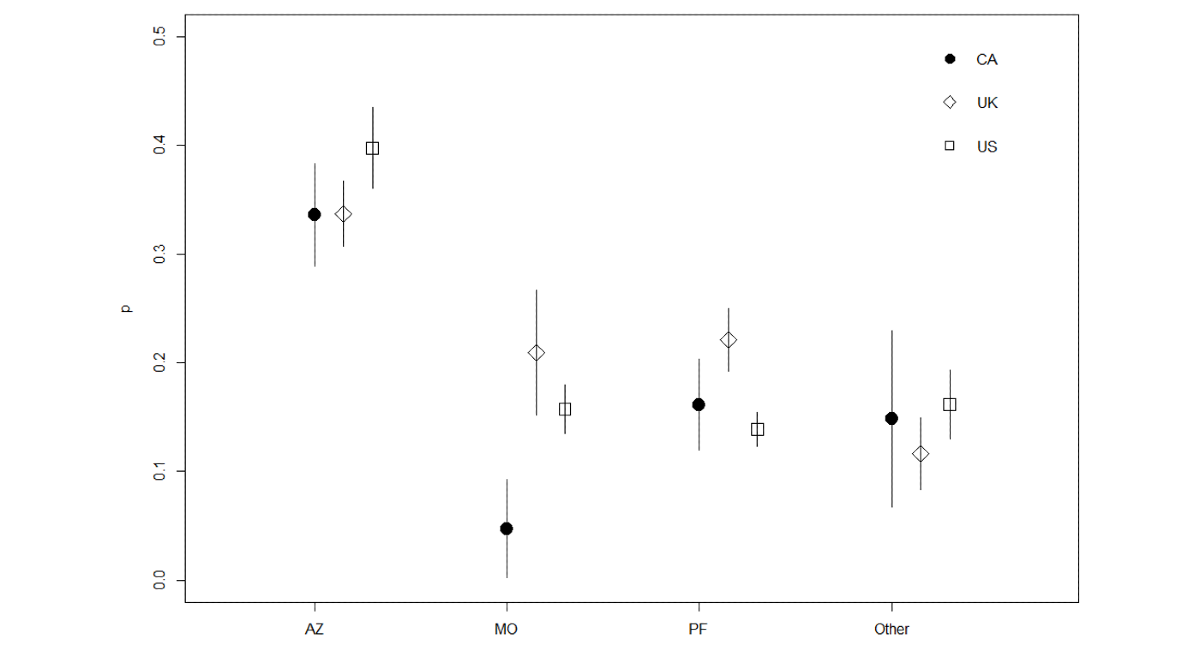
Proportion of NVI tweets by vaccine developer and country. AZ: Oxford/AstraZeneca; CA: Canada; MO: Moderna; NVI: negative vaccine information; Other: any other COVID-19 vaccine developer; PF: Pfizer-BioNTech; UK: United Kingdom; US: United States.

Comparisons of NVI tweets across different vaccine developers are shown on Figure 2. The number of tweets varied by developer, but the most noteworthy contrast involved Oxford/AstraZeneca, for which NVI tweets made up almost 35.69% (713/1998) of the content, more than double the percentage of NVI tweets observed for other developers (Moderna: 204/1290, 15.81%; Pfizer-BioNTech: 477/2920, 16.34%; Other: 139/967, 14.37%; *P*<.001 for all pairwise comparisons of Oxford/AstraZeneca with other developers). Figure 3 provides more detail with the percentage of NVI tweets by country and vaccine developer. NVI tweets were more commonly associated with the Oxford/AstraZeneca vaccine than the other vaccine developers for tweet authors in all 3 counties studied. The figure also suggests that a higher proportion of NVI tweets related to Moderna and Pfizer-BioNTech originated in the United Kingdom than in the United States or Canada.

**Figure 4:**
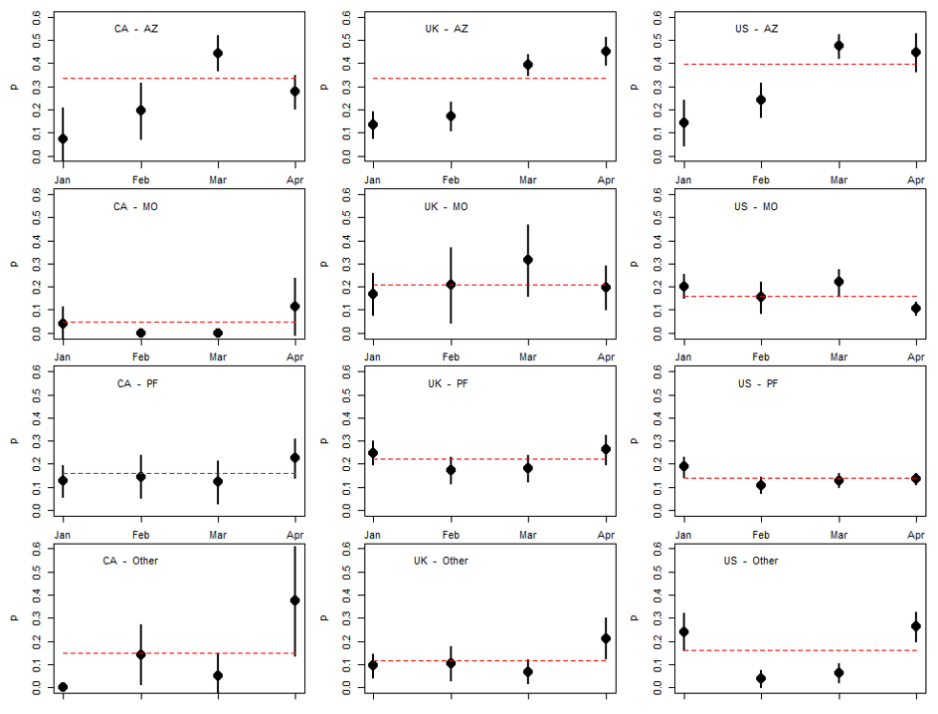
Proportion of NVI tweets by month, country and vaccine developer. Vertical lines are 95% CIs. AZ: Oxford/AstraZeneca; CA: Canada; MO: Moderna; NVI: negative vaccine information; Other: any other COVID-19 vaccine developer; PF: Pfizer-BioNTech; UK: United Kingdom; US: United States.

Figure 4 illustrates the proportion of NVI tweets by country, vaccine developer, and month of year. The dotted horizontal lines are the proportions of NVI tweets for the entire study period. These figures illustrate a very similar trend of rising NVI tweets over time associated with the Oxford/AstraZeneca vaccine for Twitter users in all 3 countries. Another noteworthy observation is the uniformly higher proportion of NVI tweets authored by Twitter users in the United Kingdom associated with the Pfizer-BioNTech and Moderna vaccines, although due to the smaller number of Moderna-related tweets authored by UK Twitter users, these proportions have a considerably larger confidence interval. Unlike the Oxford/AstraZeneca vaccine, neither of these observations is accompanied by a clear trend over time. In Canada, it appears that the highest volume of NVI tweets occurred in April for all vaccines.

**Table 2:** Anti-vaccine and negative vaccine information by account type.

Tweet	Government (n=140), n (%)	Media (n=1755), n (%)	Medical (n=1078), n (%)	Other (n=4032), n (%)	Closed, deleted, or restricted account (n=300), n (%)
Anti-vaccine	0 (0)	3 (0.17)	0 (0)	108 (2.68)	19 (6.33)
Negative vaccine information	19 (13.57)	342 (19.49)	81 (7.51)	1002 (24.85)	89 (29.67)

## Discussion

### Principal Findings

Our results indicate that less than 2% of vaccine-related tweets contain anti-vaccine content and 21% contain NVI. This finding suggests that very little Twitter content was anti-vaccine in early 2021, which is consistent with the findings of other research [25]. When compared to research on pre–COVID-19 anti-vaccine content on Twitter (which found that anti-vaccine content was closer to 9%) [26], this finding could suggest that the changes in policy in late 2020 did reduce anti-vaccine content. Although we found anti-vaccine content to be rare on Twitter over the study period, NVI tweets were not uncommon and were generated by a broad range of content authors. NVI content was generated by all Twitter content generator groups, making up almost 20% of the content from media sources and almost 14% of the content from government sources.

More than 25% of Twitter content authored in the United Kingdom appeared to be NVI, but in terms of absolute quantity, a plurality of NVI originated from Twitter accounts in the United States. This finding reflects one of the ongoing realities of globalized social media—that content has few barriers—and domestic regulations that attempt content control will only work if they are enforced in the jurisdictions responsible for a large share of the material. Nevertheless, it is difficult to know the reasons for the relatively low percentage of NVI tweets generated in the United States (compared to Canada and the United Kingdom). One explanation is that alternative platforms were more popular for the communication of NVI in the United States, including those with specific political agendas that emerged in the last year. As such, NVI content generators in the United States may have shifted to an alternative platform in the anticipation of changes to Twitter’s content policy, resulting in less NVI content on Twitter. It is also possible that Twitter targeted more content authored in the United States than in the United Kingdom or Canada. However, other explanations are possible, and our analysis offers no clear evidence explaining this observation.

In January, the volume of NVI tweets was similar for all vaccines, but as concerns about the safety of the Oxford/AstraZeneca vaccine rose in March of 2021, NVI tweets specific to this vaccine rose for Twitter users in all 3 countries—a finding consistent with other research [27]. Unlike the United States, both Canada and the United Kingdom approved and administered the Oxford/AstraZeneca vaccine for emergency use; however, Twitter users in the United States reported the highest proportion of NVI tweets mentioning the Oxford/AstraZeneca vaccine. Twitter users in the United Kingdom were responsible for more NVI content related to the Moderna and Pfizer-BioNTech vaccines than in Canada or the United States. This finding is noteworthy since Pfizer-BioNTech and Moderna made up a smaller quantity of vaccines acquired for use in the United Kingdom than Oxford/AstraZeneca. This pattern—where less commonly used vaccines are associated with higher NVI—could be explained by the absence of positive public health messaging related to that vaccine. In the United States, for example, public health officials and clinicians would have no reason to make Twitter posts about getting the Oxford/AstraZeneca vaccine, as it was not available for use, which could result in a smaller denominator in the calculation of NVI prevalence and a higher proportion of negative tweets associated with this vaccine.

Overall, our results suggest that a small fraction of COVID-19 vaccine–related tweets included anti-vaccine content, but NVI was relatively common. NVI was authored by all types of Twitter users and varied by geography, time, and vaccine developer. Unlike most anti-vaccine content, NVI could be viewed as a legitimate part of the pro-vaccine information narrative, since its presence may provide information consumers an increased sense of trust about the transparency of vaccine developers and government. Its presence on social media could even be promoted as evidence of an effective communication system that is honest about the potential negative effects of vaccines while promoting the overall health benefits. Indeed, the high level of NVI associated with the Oxford/AstraZeneca vaccine over time could even be seen as an important indicator of openness and transparency as evidence changes over time.

This research provides no insight as to whether NVI on Twitter has any impact on COVID-19 vaccine hesitancy. Some research has suggested that certain types of information presented in the media could increase vaccine hesitancy [7,28], yet other research has suggested that Twitter content has little effect on public opinion or behavior [29,30]. Arguably, if NVI on Twitter or other forms of media is a concern, it is not through its presence but the absence of context required for proper interpretation. Information about adverse reactions is not by itself evidence against the benefits of vaccination, but without context for understanding the balance of risks, it could cause concern that creates or amplifies vaccine hesitancy [16]. Research in risk communication suggests the importance of a foundational knowledge of science and numeracy [31]. Since the availability of NVI is likely to persist in all media, efforts must continue to improve how information is communicated by focusing on individualized risk estimates and visual risk displays [32].

### Implications

Content moderation remains a challenge for all media platforms, but unlike most traditional media, social media content is user generated, with the social media exerting little editorial control. Changes in policy in 2020 seem to have impacted the content on social media, but striking the right balance between freedom of expression and content control remains an important challenge. Further discussion of the content moderation process is a critical public service and can help us better understand the social media platforms we use [33].

Research conducted in the early phase of the COVID-19 outbreak [34] had suggested a substantial rise in anti-vaccine content on Twitter even before vaccines were widely available. After the changes in COVID-19–related policy, some Twitter users were banned and some of their content was removed. As reported in recent research, some censored content authors view the censorship as a sign of subterfuge and that social media companies are complicit in a cover-up of the true harms of vaccination [35]. We found that anti-vaccine information was rare on these platforms as the vaccines were rolled out to the public; however, critiques that all negative information about vaccines has been suppressed is not consistent with the evidence presented in this study. In the early days of vaccination, Twitter was widely used as a platform for sharing information about adverse events associated with vaccination, including content published by official public health sources as well as the media. Moreover, NVI associated with the Oxford/AstraZeneca vaccine is consistent with the general concern that it was associated with more adverse reactions in early 2021, something that would not have been expected if Twitter had universally censored the NVI that could harm the reputation of vaccine manufacturers.

Nonetheless, the presence of NVI may still present a challenge to public health communicators if it results in a net increase in vaccine hesitancy. NVI may underlie several cognitive biases that contribute to vaccine hesitancy [36]. Personal stories about adverse reactions can create a negative impression of the vaccination experience that is easily recalled when making decisions—a form of availability bias [37]. As a social media platform, Twitter is particularly effective at delivering short, easily digested, and impactful messages rather than scientifically informed and data-driven arguments. Early negative impressions about vaccines that were neither anti-vaccine nor misinformation may have had a substantial influence on the prevalence of vaccine hesitancy, particularly in early 2021.

Given that NVI is common and can be viewed as a normal part of the health communication process, eliminating it is neither possible nor desirable. Growing evidence shows that personal narratives (from experts and nonexperts) are effective at engaging social media consumers about health information and may often be more effective than strictly informational guidance [38,39]. On this basis, countering the effects of NVI on vaccine hesitancy may be best addressed on Twitter by offering alternative positive personal narratives about pro-vaccine experiences [40]. Existing research suggests that such pro-vaccination narratives may be more effective when accompanied by video or audio content rather than text alone [41], but further works needs to be done to determine how these messages can be used most effectively.

### Limitations

One important limitation to this study is the lack of agreement on anti-vaccine tweets, which had, at best, fair interrater agreement [42]. The text limit for individual tweets can make the meaning and intent of a tweet difficult to interpret, and determining intent is important for classifying tweets as anti-vaccine. It is for this reason that an extensive analysis of anti-vaccine tweets was not presented. Importantly, however, coding did not yielded a large number of anti-vaccine tweets—with 3 of the 4 coders yielding less than 2% of their tweets as anti-vaccine. The share of NVI tweets was similarly uniform, although interrater agreement was not particularly high.

The search criteria used to select tweets for analysis were likely to have excluded relevant tweets from the sampling frame. First, we did not include alternative spellings of vaccine that are sometimes used by the anti-vaccine community. This exclusion very likely led to an underestimation of anti-vaccine tweets in the sampling frame. It is difficult to estimate the effect of excluding these search terms on our analysis, but even if we underestimated by half, it would still leave less than 4% of the tweets as anti-vaccine and would not dramatically change our conclusions. Second, the georeferencing process eliminated a large number of tweets, and it is unclear if this exclusion introduced a bias into the results. It is possible that certain forms of geographic identification that we did not consider—for example, referring to the city a person lives in rather than the country or province/state—may be associated with disposition toward vaccines in some way. Although the authors cannot rule out this possibility, it seems implausible that such an effect would have a large impact in all 3 jurisdictions studied, and it seems reasonable to assume this effect would be small.

### Conclusions

Our results suggest that Twitter was not a substantial source of anti-vaccine content in early 2021, but it still contained a large quantity of information that could contribute to vaccine hesitancy. It is important to note, however, that NVI is not unique to social media and can be found in traditional media sources and even public health notifications from government agencies. Therefore, it would be inappropriate to treat all (or even most) NVI as socially deleterious. Moreover, this information (particularly when authored by reputable sources) may have the long-term benefit of increasing trust in public health messaging, as open communication of negative and positive effects could contribute to increase faith in the transparency and honesty of public health messaging.

## Abbreviations

NVI: negative vaccine information
